# Oral health status and behaviours of preschool children in Hong Kong

**DOI:** 10.1186/1471-2458-12-767

**Published:** 2012-09-11

**Authors:** Chun-Hung Chu, Ping-Lit Ho, Edward CM Lo

**Affiliations:** 1 Faculty of Dentistry, The University of Hong Kong, Hong Kong SAR, China; 2 University Health Service, The University of Hong Kong, Hong Kong SAR, China

**Keywords:** Dental caries, Oral hygiene, Oral health, Toothbrushing, Preschool children, Hong Kong, China

## Abstract

**Background:**

Dental caries is a major public health problem in many countries. Since the last territority-wide dental survey of Hong Kong preschool children was conducted in 2001, a survey to update the information is necessary. This study aimed to describe the dental caries experience of preschool children in Hong Kong and factors affecting their dental caries status.

**Methods:**

A stratified random sample of children from seven kindergartens in Hong Kong was surveyed in 2009. Ethical approval from IRB and parental consent was obtained. Clinical examinations of the children were performed by two calibrated examiners using disposable dental mirrors, an intra-oral LED light and ball-ended periodontal probes. A questionnaire to investigate possible explanatory factors for caries status was completed by the children’s parents. Caries experience was recorded using the dmft index. Multifactor-ANOVA was used to study the relationship between dental caries experience, and the background and oral health-related behaviours of the children.

**Results:**

Seven hundred children (53% boys), mean age 5.3 ± 0.7 years were examined. The mean dmft score of the surveyed children was 2.2 and 51% of them had no caries experience (dmft = 0). Most (>95%) of the decayed teeth were untreated. Statistically significant correlations were found between dental caries experience of the children and their oral health-related habits, family income, parental education level and parental dental knowledge.

**Conclusions:**

Early childhood dental caries was prevalent among the preschool children in Hong Kong. Their caries experience was associated with their oral health-related behaviours, socio-economic background, and parental education and dental knowledge.

## Background

Dental caries is a major public health problem affecting most of the children in many countries world-wide [1,2]. Dental caries is also prevalent among preschool children in China. In the second national oral health survey conducted in 1995, it was found that 77% of the 5-year-old children in China were affected by dental caries and the mean dmft score was 4.5 [3]. There has been a decline in dental caries among the children in the industrialized countries in the latter half of the last century [4]. However, there are also epidemiological findings which show that the magnitude of change varies in different child populations and the decline may come to an end when low levels of prevalence in dental caries are reached [5]. A recent review of epidemiological data by Bagramian et al. [6] found caries prevalence among children increased in many developed and developing countries. Untreated tooth decay will cause pain, form dental abscess, and lead to severe local and systemic infections [7]. This increase in dental caries signals a pending public health crisis to many communities.

Over the years, a number of preventive programs have been implemented by the government of Hong Kong to reduce dental caries in the children. Water fluoridation has been implemented in Hong Kong since 1961 and the caries experience of the children has reduced [8,9]. In addition, an oral health education program for preschool children has been carried out since 1993, serving to promote good oral health-related behaviours among the children attending kindergartens and to increase the kindergarten teachers’ and parents’ oral health knowledge. Apart from carrying out preventive measures, the School Dental Care Service (SDCS), established in 1980, also provides basic curative treatments for children attending primary schools in Hong Kong. However, preschool children are not eligible to enroll in this service, and most of them can only seek care from private dentists. Past survey in Hong Kong found that the preschool children who experienced dental caries were often from the poor and disadvantaged families, and private dental services were often unaffordable for them [8]. The caries experience among young children in Hong Kong remains high in the past decade. It is unknown if the high caries prevalence of children is the results of the development in Hong Kong or caused by the incoming of Chinese immigrants. Implementations of further preventive measures, such as extending the SDCS to cover the preschool children, are necessary to improve the dental caries situation.

In order to better understand the dental health of preschool children in Hong Kong, a new survey on a representative sample is warranted. The collected information would be useful to the dental profession and the government in Hong Kong for planning dental services for young children. Additional information on the children’s oral health-related behaviours, and their parents’ oral health attitudes and knowledge would be needed for further development of oral health education programs. The aim of this study was to obtain updated information on the dental caries situation of preschool children in Hong Kong and the factors that affected their caries status.

## Methods

### Selection of children and sample size

This study was approved by the Institutional Review Board of the University of Hong Kong (IRB 09–302). It was carried out in March 2009. Stratified random sampling was employed to select kindergartens from a list of registered kindergartens according to the proportion of kindergartens in Hong Kong’s three main geographical areas, namely Hong Kong Island, Kowloon, and the New Territories [10]. All seven selected kindergartens agreed to participate in this study. Oral health education was given to the preschool children prior to the survey. All children attending the last two years of the preschool program in the selected kindergartens were invited to participate in this study. A letter was sent to the children’s parents informing them of the study and to ask for their consent. Children with serious general health problems, such as congenital heart disease, were excluded from the study.

Sample size calculation was performed with reference to a previous survey of preschool children in Hong Kong which reported a mean dmft score of 1.6 ± 2.9 [8]. The 95% confidence interval was set to be 0.15 on both sides of the mean dmft score. The computed minimum sample size was 495. Assuming a response rate of 80%, the number of preschool children to be invited would be at least 619.

### Questionnaire survey

A visit was paid to each kindergarten before the survey to deliver the questionnaires and to discuss the protocol with the kindergarten teachers. The parents were asked to complete a questionnaire specially designed for this study. The questionnaire consisted of four parts (Attached as Additional file 1) and the following information was collected:

1) The child’s personal data - sex, age, place of birth;

2) The child’s socio-economic background - parents’ education level and family income;

3) The child’s oral health-related behaviours – snacking habit, frequency of toothbrushing, brushing with or without adult assistance, and dental visit pattern; and

4) Parent’s dental knowledge.

To assess the parents’ dental knowledge, there were 21 multiple choice questions in the questionnaire on the causes and prevention of dental diseases [8]. One score was given to each correct answer; and no score was given to a wrong or ‘don’t know’ answer. Thus, the total dental knowledge score could range from 0 to 21. The parents were then categorized into three groups according to their dental knowledge scores in 3 equal intervals - low (scored 0–7), middle (scored 8–14), and high (scored 15–21).

### Clinical examination

The clinical examinations of the preschool children were carried out by two trained and calibrated examiners using disposable dental mirrors, intra-oral LED lights, and ball-ended WHO CPI probes. Diagnosis of dental caries was made according to the criteria recommended by the World Health Organization [11], i.e. when a lesion in a pit or fissure, or on a smooth tooth surface, had an unmistakable cavity, undermined enamel or a detectably softened floor or wall. The dmft index was used to record the dental caries experience of the primary dentition of the children: ‘d’ stands for decayed tooth, ‘m’ denotes missing tooth due to decay, and ‘f’ represents filled tooth. Calibration of examiners with an experienced oral epidemiologist was carried out on five child patients of a dental hospital prior to the survey. Duplicate examinations were carried out on 10% of the children during the survey. Kappa statistic was used to assess the inter-examiner reproducibility.

### Statistical analysis

Data analysis was performed using the software Statistical Package for Social Sciences version 17.0 (SPSS Inc., Chicago, Illinois, USA). T-test and one-way ANOVA were used to assess the statistical significance of the differences in dental caries experience (dmft score) found between groups. Bonferroni comparison was used to compare the groups when the independent variable was found to be a significant factor affecting the caries experience. Multi-factor ANOVA was used to investigate the effects of the independent variables studied, including the demographic and socio-economic background of the child and the child’s oral health-related behaviours, on the child’s dental caries experience. The dependent variable was the child’s dmft score and all the independent variables were entered into the model. A backward stepwise procedure was used to remove the variables that were not statistically significant. The final model only contained variables that were statistically significant. The level of statistical significance for all tests was set at 0.05.

## Results

A total of 764 kindergarten children from 7 kindergartens were invited to participate in this study. In 54 cases, either the consent forms were not returned or the returned questionnaires were incomplete. Ten children were excluded from the study because they were too uncooperative to be examined or had serious general health problem. Thus, 700 preschool children were finally examined. The response rate was 92% (700/764). The value of Kappa statistic for diagnosis of tooth status was 0.95.

The mean age of the 700 surveyed children was 5.3 ± 0.7 years. Approximately half (53%) of them were boys. The proportion of children with no dental caries experience (dmft = 0) was 51% while 7% of the children had a dmft score ≥8. The mean dmft score of the children was 2.2 ± 3.5 (Table 1). Over 95% of the mean dmft score was contributed by untreated decayed teeth (dt = 2.1). The mean number of missing or filled primary teeth due to caries was very small (mt < 0.1; ft = 0.1).

**Table 1:** Dental caries experience of the surveyed children according to gender, age and place of birth

**Independent variable**	**Group**	**N**	**% dmft > 0**	**dmft ± S.D.**	**Significance**
	All	700	49	2.2 ± 3.5	
Gender	Boys	373	50	2.4 ± 3.7	p > 0.05
	Girls	327	48	2.1 ± 3.3	
Age	4	239	41	1.9 ± 3.4	p > 0.05
	5	338	48	2.3 ± 3.6	
	6	123	64	2.6 ± 3.3	
Place of birth	Hong Kong ^a^	649	48	2.2 ± 3.5	p = 0.02
	China ^b^	42	69	3.5 ± 4.0	

There is no statistically significant difference between the mean dmft score of the boys and that of the girls (p > 0.05). The percentage of children with caries experience gradually increased with age, from 41% at age 4, through 48% at age 5, to 64% at age 6 (Chi-square test, p < 0.01). Children born in Mainland China had a higher mean dmft score than that of children born in Hong Kong (3.5 vs. 2.2, p = 0.02).

**Figure 1:**
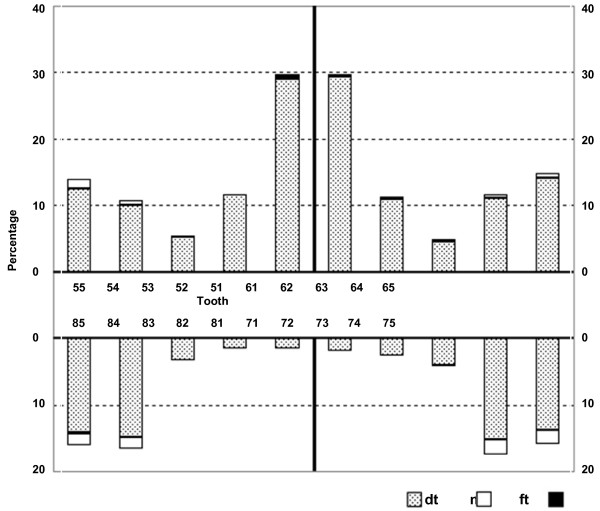
Distribution of caries experience of children (N = 700).

The prevalence of dental caries experience was higher among the maxillary teeth than their mandibular counterparts (Figure 1). In the maxillary dental arch, canines had the lowest caries prevalence, while in the mandibular dental arch, incisors and canines had the lowest caries prevalence. Maxillary incisors had a higher caries prevalence than the mandibular incisors (29% vs. 2%), while the prevalence among the maxillary and the mandibular molars was similar. Slightly more than one third (36%) of the children had caries in their maxillary anterior teeth and this was associated with the presence of caries in their posterior teeth (p < 0.01).

In the bivariate analysis, duration of bottle feeding was found to have no statistically significant association with the child’s dental caries experience (p > 0.05) while children who took sweet snacks twice or more a day had a higher mean dmft score than that of children who did not (2.5 vs. 2.0, p = 0.05) (Table 2). In relation to the children’s oral hygiene practices, children who started toothbrushing by 12 months of age had lower dental caries experience than those who did not (p = 0.01). There were no statistically significant associations between the children’s dental caries experience and their frequency of toothbrushing or parental assistance in toothbrushing. A higher mean dmft score was found among the children who had visited a dentist compared to that of children who had not (3.1 vs. 2.0, p < 0.01).

**Table 2:** Mean dmft scores of the surveyed children according to their dietary and oral hygiene and dental visit behaviours

	**Group**	**N (%)**	**dmft ± S.D.**	**Sig.**	**Bonferroni Comparison**
Dietary habits					
Bottle feeding	Yes	143 (20%)	2.3 ± 3.4	p > 0.05	
	No	557 (80%)	2.2 ± 3.5		
Bottle feeding duration (months)	≤ 24	202 (29%)	2.5 ± 4.0	p > 0.05	
	> 24	495 (71%)	2.1 ± 3.3		
Snacking frequency (times per day)	≤ 2	386 (55%)	2.0 ± 3.3	p = 0.05	
	> 2	314 (45%)	2.5 ± 3.7		
Oral hygiene habits					
Age toothbrushing started (months)	1 – 12 ^a^	84 (12%)	1.1 ± 2.2	p = 0.01	a < c, d
	13 – 18 ^b^	172 (25%)	2.1 ± 3.5		
	19 – 24 ^c^	160 (23%)	2.5 ± 3.7		
	> 24 ^d^	284 (41%)	2.5 ± 3.7		
Brushing frequency (times/day)	< 2	175 (25%)	2.6 ± 3.7	p > 0.05	
	≥ 2	525 (75%)	2.1 ± 3.5		
Parental assistance in toothbrushing	Yes	559 (80%)	2.2 ± 3.5	p > 0.05	
	No	141 (20%)	2.5 ± 3.6		
Use other cleansing aids (N = 700)	Yes	156 (22%)	2.5 ± 3.7	p > 0.05	
	No	544 (78%)	2.1 ± 3.4		
Dental service use					
Visited a dentist	Yes	88 (13%)	3.1 ± 3.7	p < 0.01	
	No	583 (83%)	2.0 ± 3.5		

It was found that children whose parents had better dental knowledge had lower caries experience (p < 0.01). Children who came from families with a higher income, or whose parents had a higher education level also had less dental caries (Table 3). Furthermore, children from single-parent families were found to have more dental caries. Children living in families which employed a domestic helper to look after the child had a lower dental caries experience.

**Table 3:** Mean dmft scores of the surveyed children according to parental dental knowledge and socio-economic status

**Independent variable**	**Group**	**No. (%)**	**dmft ± S.D.**	**Sig.**	**Bonferroni Comparison**
Parental dental knowledge	Score 0-7^a^	69 (10%)	3.0 ± 4.2	p < 0.01	a, b > c
	Score 8-14^b^	358 (51%)	2.7 ± 3.8		
	Score 15-21^c^	273 (39%)	1.4 ± 2.6		
Family income (HKD)	≤ 10000 ^a^	234 (33%)	2.8 ± 4.0	p < 0.01	a, b > c
	10000 – 20000 ^b^	241 (34%)	2.6 ± 3.6		
	≥ 20000 ^c^	225 (32%)	1.2 ± 2.5		
Father’s education level	Primary or below ^a^	93 (14%)	2.7 ± 4.1	p < 0.01	a, b > c
	Secondary ^b^	477 (69%)	2.4 ± 3.6		
	Tertiary or above ^c^	136 (20%)	1.1 ± 2.1		
Mother’s education level	Primary or below ^a^	125 (18%)	2.9 ± 4.1	p < 0.01	a > c
	Secondary ^b^	477 (69%)	2.2 ± 3.4		
	Tertiary or above ^c^	92 (13%)	1.3 ± 2.6		
Parenthood	Single	31 (4%)	3.8 ± 4.6	p = 0.01	
	Double	669 (96%)	2.1 ± 3.4		
Care taker	Parents ^a^	469 (67%)	2.2 ± 3.5	p < 0.01	a, b, d > c
	Grandparents ^b^	112 (16%)	3.0 ± 4.1		
	Domestic helpers ^c^	105 (15%)	1.2 ± 2.2		
	Others ^d^	14 (2%)	3.2 ± 3.7		

Results of the multivariate analysis show that four independent variables remained in the final model and these had a statistically significant correlation with the children’s dental caries experience, namely the child’s snacking frequency, dental attendance, parental dental knowledge and family income (Table 4).

**Table 4:** Relationship between dental caries experience of the surveyed children and selected independent variables (final model of multi-factor ANOVA) (N = 700)

**Independent variable**	**Group**	**Beta**	**SE (Beta)**	**Significance**	**Bonferroni Comparison**
Snack frequency (times per day)	≥2	0.54	0.26	p = 0.04	
	<2 ^#^				
Visited a dentist	Yes	1.47	0.35	p < 0.01	
	No ^#^				
Parental dental knowledge	Score 0-7^a^	1.24	0.46	p < 0.01	c < b, a
	Score 8-14^b^	1.02	0.28		
	Score 15-21^c #^				
Family income (HKD)	≤ 10000 ^a^	1.54	0.33	p < 0.01	c < b, a
	10000 – 20000 ^b^	1.33	0.32		
	≥ 20000 ^c #^				

F-value = 11.14; df = 6; p < 0.001; R^2^ = 0.088, # Reference category.

## Discussion

This study employed a random stratified cluster sampling technique. In theory, clusters of children should be as heterogeneous as possible and each cluster of children should be, to some extent, representative of the general child population. In this survey, the study children were chosen based on kindergartens, and the use of this sampling technique helped to reduce the time and cost associated with the survey. However, the background of the children within a cluster could be fairly homogeneous while the clusters might vary from one another. Therefore, the variance of this cluster sample with a defined sample size may be larger than that of a simple random sample, and hence the estimates are less precise. Despite this, this study achieved a very high response rate from the children and their parents. This is partly because initial contacts were made three months in advance to allow ample time for the selected kindergartens to prepare. There were pre-survey visits to the kindergartens and invitation letters, consent forms, and questionnaires were delivered to parents through the kindergartens and sufficient time was provided for collection. In addition, there was sufficient training of and calibration exercises for the examiners before the survey, leading to a very good inter-examiner agreement.

The mean dmft score of the children surveyed in this study, 2.2, is similar to those of the 5-year-old children in previous surveys conducted in 1997 and 2001, which are 1.8 and 2.3, respectively [8,12]. There is no clear trend of change in the dental caries status of the preschool children in Hong Kong over the past decade. The findings in all the surveys that over 90% of the decayed primary teeth in the Hong Kong preschool children were untreated indicate that there has been no significant improvement in their access to and use of proper dental care services.

The dental caries experience of the preschool children in this survey is higher than that of the preschool children in a recent survey conducted in Singapore which found a mean dmft score of 1.5 [13]. It should be noted that Hong Kong and Singapore are similar in term of economic development and that both cities have implemented water fluoridation. The mean dmft score of the 5-year-old children in recent survey conducted in Taiwan was 7.0 [14] and that found in the second national oral health survey conducted in Mainland China was 4.5 [3]. When compared to these figures, the dental caries experience of the Hong Kong preschool children is much lower.

In the United Kingdom where the National Health Service provides free comprehensive dental care for preschool children, a mean dmft score of 1.6 for 5-year-old children was reported in 2003 [15]. In New South Wales in Australia where there is a health program for preschool children which incorporates components of oral health education, risk assessment and clinical care, a mean dmft score of 1.5 was reported [16].

There are a number of meaningful observations in this study. Dental caries starts at an early age among the preschool children in Hong Kong, as shown in the finding that two fifths of the surveyed children at age four had dental caries, and becomes more severe over time. There is a need to implement dental caries prevention measures at early age and to reinforce them continuously. Different oral health promotion measures may be needed because early childhood dental caries is a multi-factorial disease [17].

The observed dental caries pattern in this study sample agrees with the common observations and general theories [7]. The highest prevalence of caries was found in the maxillary incisors. This can be explained by the longer duration of exposure to cariogenic challenge according to the chronological tooth eruption sequence. It has been shown that the prevalence of caries in posterior primary teeth is higher in children who have caries in their maxillary anterior teeth [18]. This was also found in this study among the preschool children in Hong Kong. Presence of caries in maxillary primary anterior teeth should be noted during dental examination of young children and used as an indicator for high caries risk in the posterior teeth, and these children should receive more preventive dental care.

Results of this study show a correlation between dental caries of the Hong Kong preschool children and their snacking habit which is in agreement with those of other studies [8,17]. A high frequency of sugar intake prolongs the duration of lowered pH in the mouth which leads to a higher demineralization rate resulting in dental caries [19]. Thus, the control of frequent sweet snack taking in young children is important in preventing dental caries.

Findings of this study show that toothbrushing is another important factor affecting dental caries in young children. The surveyed children who had their teeth brushed at an early age had less dental caries. This has also been found in earlier studies conducted in Ireland [20] and in China [21]. It should be noted that eruption of primary teeth into the mouth starts before 12 months of age and continues till 2–3 years. Practice of toothbrushing can help to keep the erupted teeth clean as well as to deliver fluoride onto the tooth surfaces if fluoridated toothpaste is used. Promotion of parental and self toothbrushing in young children should be carried out in the maternity and child health centres in Hong Kong where health care services for infants and health education for parents are delivered.

The finding that among the preschool children surveyed in this study, those who had visited a dentist had more dental caries experience needs some explanation. A possible reason is that for most of the young children Hong Kong, they are only brought to see a dentist when they have dental problems such as tooth decay. This may be related to the prevalent problem-oriented dental care seeking behavior among the adults in Hong Kong [22]. To change this pattern of dental care seeking behavior, more effort should be made to promote early and regular preventive dental visits among young children. The establishment of a public or a subsidized dental service for the preschool children in Hong Kong would probably help to improve the situation.

Another group of factors which have significant associations with the study children’s dental caries experience is their demographic and socio-economic background. Similar to the findings of previous survey of preschool children in Hong Kong [8], children born in Mainland China had more dental caries than those born in Hong Kong. Since there has been a recent sharp increase in the number of new Chinese immigrant children in Hong Kong (Census and Statistics Department, [23]), it is likely that the dental caries situation of the preschool children in Hong Kong will become worse if no actions are taken.

Similar to other studies on young Chinese children [24,25], this study found the parents’ dental knowledge to have a significant association with their child’s dental caries experience. In this study, less than 40% of the parents could correctly answer two thirds or more of the dental knowledge questions in the questionnaire. Quite a large proportion of the parents in Hong Kong may have a variety of misconceptions on matters relating to the dental health of their young children. Dental preventive measures for preschool children would probably be more successful when all three parties concerned, namely children, teachers and parents, cooperate with one another. Oral health promotion programs should include educating the parents so as to improve their dental health knowledge. Hence, besides involving the kindergarten teachers, more effort should be paid to involve the parents as well in oral health programs for preschool children in Hong Kong.

Social inequality in dental health among children exists in many countries [26-28]. This was also found in this study among the preschool children in Hong Kong. Children from the lower socio-economic classes as indicated by a lower family income and lower parental education level had more dental caries. The finding that the study children who were taken care of by domestic helpers had less dental caries may be because these children came from wealthier families that could afford to employ domestic helpers. To improve social equality in oral health, the Hong Kong government working with the dental profession should allocate more resources for providing oral health promotion activities and services for the young children who are less privileged so as to improve their dental health-related behaviours and conditions.

## Conclusion

Dental caries is common among the preschool children in Hong Kong with around half of them having untreated carious teeth and their dental caries experience increases with age. The children’s dental caries situation is related to their toothbrushing and snacking habits, as well as to their socio-economic background.

## Competing interests

The authors declare that they have no competing interests.

## Authors’ contributions

PL Ho: conduct dental examination, analyze data, and write the manuscript. CH Chu: design and implement study, training examiners, analyze data, and write the manuscript. ECM Lo: check data analysis and write the manuscript. All authors read and approved the final manuscript.

## Pre-publication history

The pre-publication history for this paper can be accessed here:

http://www.biomedcentral.com/1471-2458/12/767/prepub

## Supplementary Material

Additional file 1 Questionnaire (Chinese). Click here for file
