# Supplementary material for: Oral health status and behaviours of preschool children in Hong Kong
Source: BMC Public Health. 2012 Sep 11;12:767. doi: 10.1186/1471-2458-12-767 (PMC3490858; doi:10.1186/1471-2458-12-767)
Supplement: Additional file 1 — Questionnaire (Chinese). [file 1471-2458-12-767-S1.pdf]

Questionnaire (Chinese)

甲部：子女的飲食習慣

1. 你的子女現在有否用奶瓶含奶或甜味飲品的睡覺習慣？
   ☐ 有　　☐ 沒有

2. 你的子女在何時停止使用奶瓶餵奶及其他甜味飲品：
   ☐ 1 至 12 個月
   ☐ 13 至 18 個月
   ☐ 19 至 24 個月
   ☐ 24 個月後
   ☐ 其他，請註明：___________________

3. 除正餐以外，你的子女平均每天進食多少次？（包括汽水、果汁、零食、糖果、餅乾、水果、甜品等）
   ☐ 沒有
   ☐ 1 - 2 次
   ☐ 3 - 4 次
   ☐ 5 - 6 次
   ☐ 7 - 8 次
   ☐ 9 - 10 次
   ☐ 10 次以上

乙部：子女的口腔衛生習慣

4. 你的子女何時開始刷牙：
   ☐ 1 至 12 個月
   ☐ 13 至 18 個月
   ☐ 19 至 24 個月
   ☐ 24 個月後
   ☐ 其他，請註明：___________________

5. 你的子女現時每日刷牙次數：
   ☐ 不刷牙　☐ 間中　☐ 每天 1 次　☐ 每天 2 次 或 以上

6. 有沒有旁人協你的子女刷牙：
   ☐ 有　☐ 沒有

7. 你有否帶子女看過牙醫:
   ☐有，定期檢查　☐ 有，不定期　☐ 沒有

8. 你的子女有沒有用其他方法清潔口腔？　（例如漱口水，牙線）
   ☐ 有
   ☐ 沒有

丙部：口腔健康常識

9. 蛀牙的成因是：

		是	否	不知道
a)	吃糖太多	☐	☐	☐
b)	牙齒不潔	☐	☐	☐
c)	牙蟲侵蛀	☐	☐	☐
d)	熱氣	☐	☐	☐

10. 防止蛀牙的方法是：

		是	否	不知道
a)	服用藥物	☐	☐	☐
b)	飲涼茶	☐	☐	☐
c)	用含氟素牙膏刷牙	☐	☐	☐
d)	減少吃糖次數	☐	☐	☐

11. 氟素對牙齒的影響是：

		是	否	不知道
a)	沒有影響	☐	☐	☐
b)	防止蛀牙	☐	☐	☐
c)	潔白牙齒	☐	☐	☐
d)	防止牙周病	☐	☐	☐

12. 以下食物會引起蛀牙

		是	否	不知道
a)	汽水	☐	☐	☐
b)	雪糕	☐	☐	☐
c)	芝士	☐	☐	☐
d)	花生	☐	☐	☐

13. 牙齒出血的成因是：

		是	否	不知道
a)	牙齒不潔	☐	☐	☐
b)	牙肉間中出血是正常的	☐	☐	☐

14. 防止牙周病的方法是：

		是	否	不知道
a)	刷牙以保持口腔清潔	☐	☐	☐
b)	鹽水漱口	☐	☐	☐
c)	定期洗牙	☐	☐	☐

丁部：學生資料

15. 學生姓名：_________________

16. 性別：☐ 男　　☐ 女

17. 出生日期：____________年___________ 月

18. 出生地點：☐ 香港　☐ 中國大陸　☐ 其他，請註明：___________________

19. 聯絡電話：______________________ (以便日後跟進工作)

戊部：其他資料

20. 閣下的子女是生長在：
    - ☐ 單親家庭
    - ☐ 雙親家庭

21.閣下的家庭總收入：
    - ☐ HK $ 10, 000 或 以下
    - ☐ HK $ 10, 001 - 15,000
    - ☐ HK $ 15, 001 - 20,000
    - ☐ HK $ 20, 001 - 25,000
    - ☐ HK $ 25, 001 - 30,000
    - ☐ HK $ 30, 001 - 35,000
    - ☐ HK $ 35, 001 或 以上

22. 家長的教育程度：

	父	母
小學程度或以下	☐	☐
中學程度及預科	☐	☐
大專程度 或 以上	☐	☐

23.你的子女通常是由誰照顧？（請選其一）
    ☐ 父母　☐ 祖父母　☐ 家傭　☐ 其他 (如親戚朋友)

~ 多謝 ~

Questionnaire (English)

Part A: Eating Habit of Your Children

1. Is your child currently bottle-fed with milk or sugary drinks before sleeping?
   ☐ Yes ☐ No

2. When does your child stop bottle-feeding with milk or sugary drinks?
   ☐ 1 to 12 months old
   ☐ 13 to 18 months old
   ☐ 19 to 24 months old
   ☐ After 24 months old
   ☐ Others, please specify: ___________________

3. Other than meals, how often does your child snack? (e.g., soft drinks, juice, snack, candy, biscuits, fruits, desserts)
   ☐ None
   ☐ 1 - 2 times
   ☐ 3 - 4 times
   ☐ 5 - 6 times
   ☐ 7 - 8 times
   ☐ 9 - 10 times
   ☐ More than 10 times

Part B: Oral Hygiene Habit of Children

4. When did your child start brushing teeth?
   ☐ 1 to 12 months old
   ☐ 13 to 18 months old
   ☐ 19 to 24 months old
   ☐ After 24 months old
   ☐ Others, please specify: ______________

5. How many times does your child brush everyday?
   ☐ Never ☐ Seldom ☐ Once ☐ Two times or more

6. Does anyone assist your child in brushing his/her teeth?
   ☐ Yes ☐ No

7. Have your child ever seen a dentist?
   ☐ Yes, with regular review ☐ Yes, without regular review ☐ Never

8. Does your child use other aids to clean his/her teeth? (e.g., mouth-rinse, dental floss)
   ☐ Yes ☐ No

Part C: Oral Health Knowledge

9. The causes of dental decay include:

	Yes	No	I don't know
a) Too much consumption of candies	☐	☐	☐
b) Unclean teeth	☐	☐	☐
c) Tooth worms attack	☐	☐	☐
d) "Hot air"	☐	☐	☐

10. Preventions of tooth decay include:

	Yes	No	I don't know
a) Medicine	☐	☐	☐
b) Herbal tea	☐	☐	☐
c) Use of fluoridated toothpaste	☐	☐	☐
d) Decrease frequency of sugar consumption	☐	☐	☐

11. Effects of fluoride to teeth include:

	Yes	No	I don't know
a) No effect	☐	☐	☐
b) Prevent tooth decay	☐	☐	☐
c) Tooth whitening	☐	☐	☐
d) Prevent periodontal disease	☐	☐	☐

12. Which of the following food can cause tooth decay?

	Yes	No	I don't know
a) Soft drinks	☐	☐	☐
b) Ice-cream	☐	☐	☐
c) Cheese	☐	☐	☐
d) Peanuts	☐	☐	☐

13. The causes of gum bleeding include:

	Yes	No	I don't know
a) Unclean teeth	☐	☐	☐
b) It is a normal phenomenon	☐	☐	☐

14. Methods to prevention of periodontal disease include:

	Yes	No	I don't know
a) Tooth brushing	☐	☐	☐
b) Saline mouth-rinsing	☐	☐	☐
c) Regular scaling (professional tooth cleaning)	☐	☐	☐

Part D: Student Information

15. Name of Student: _________________

16. Gender : ☐ M ☐ F

17. Date of birth : ____/____/______ (DD / MM / YYYY)

18. Place of birth : ☐ Hong Kong ☐ China ☐ Others, please specify: _________

19. Contact number : ________________ (for follow-ups)

Part E: Other Information

20. Home situation:
   - ☐ Both parents
   - ☐ Others

21. Monthly family income:
   - ☐ HK $ 10,000 or below
   - ☐ HK $ 10,001 - 15,000
   - ☐ HK $ 15,001 - 20,000
   - ☐ HK $ 20,001 - 25,000
   - ☐ HK $ 25,001 - 30,000
   - ☐ HK $ 30,001 - 35,000
   - ☐ HK $ 35,001 or above

22. Parent education level:

	Father	Mother
Elementary school	☐	☐
High school	☐	☐
Collage or above	☐	☐

23. Who usually take care of your child? (Please choose one)
   - ☐ Parents ☐ Grandparents
   - ☐ Domestic helper ☐ Others (e.g., relatives, friends)

~ Thank you ~
